# Papilloma of the Female Bladder

**Published:** 1890-08

**Authors:** C. D. Palmer

**Affiliations:** Cincinnati, Ohio


### PAPILLOMA OF THE FEMALE BLADDER.

BY C. D. PALMER, M. D., CINCINNATI, OHIO.

Papilloma of the Female Bladder, was the subject of a clinical lecture by Dr. C. D. Palmer.

Had three cases of this malady under his care at the Cincinnati Hospital.

Mrs. S., æt. 50, first presented herself complaining that her urine was of a pinkish tint, or, as she supposed, was bloody. Cancer of the uterus was at first suspected, but examination of this organ showed it in a healthy condition. Pressure exerted per vaginum showed the base of the bladder rather tender, but not more so than is frequently found in irritable conditions of this organ. Micturition was frequent, but not painful. The urine was slightly acid, contained much mucus, and was somewhat albuminous. The microscope showed numerous blood corpuscles, but no tube casts. The evidence in the case pointed to the bladder as the seat of the hæmaturia. The blood in the urine steadily increased in spite of ergot, iron and gallic acid. The urine, after standing a few hours, deposited great quantities of thick ropy mucus. The irritability of the bladder became more and more annoying. A growth was finally made out, which seemed to be on the anterior wall of the bladder, as the posterior wall moved freely over it. The size of the tumor was estimated to be that of a half section of an ordinary sized hickory-nut. The urethra was gradually distended with my uterine dilator till it would permit of the introduction of the smallest finger of the left hand, and afterwards the index finger of the same hand could be introduced into the bladder. The exact location of the growth was easily made out, and its growth found to be soft and friable. With the finger-nail as a curette, it was forcibly and thoroughly removed from the basement mucous membrane. The bladder was then washed out with warm water, clearing it of the blood and broken down tissue. Microscopical examination of the broken down tissue proved it to be papilloma.

Incontinence of urine continued for some 24 hours, during which time the urine was freely mixed with blood, bloodclots and broken down morbid tissue. Thereafter, the frequent micturition, the bloody urine and mucous urine rapidly abated. The general health improved very much. She was given Chian turpentine grs. ii–iii in an emulsion, and to this, so far at least as the catarrhal state of the bladder was concerned, was due much of the improvement.

Unfortunately, the relief obtained proved not to be permanent. The symptoms all gradually returned, and in less than one year were as bad as ever before. The general health also began to suffer, though it was not so much impaired as formerly. Palpation of the bladder, through the vagina, could detect no distinct tumor, though its posterior wall was thickened and indurated. The sound within the bladder revealed no special projection upon the walls, though manipulations with it were quite painful and excited considerable hemorrhage. On inspection through the meatus and through the lower urethra, now quite dilated, a highly congested and rugous condition of the mucous membranes were apparent. I now determined to repeat the operative interference, but to adopt another method. About one year after the first operation, the patient under the anæsthetic, I made an opening with a knife, guided by a sound, through the base of the bladder in the median line, enlarged the same with the scissors. The opening was made sufficiently large to admit the index finger and to permit of free exploration of the cavity of the bladder. The former site of the tumor was smooth and seemingly as healthy as any portion of the bladder to the touch. Throughout, except posteriorly, the mucous membrane was smooth, soft and velvet-like. But upon the posterior wall, around the line of incision, and anterior to this line, running forward into the urethra, numerous slight, soft, friable projections were felt. All of these were broken down and thoroughly scraped with the finger-nail. An uterine curette, with the edge somewhat sharp, was then introduced into the bladder through the urethra, and guided in its movements by the fingers of the opposite hand through the artificial opening in the bladder, I freely scraped in all directions the basement structure of all these growths. Only moderate hemorrhage followed. The bladder was washed out with the tube through the urethra with a large quantity of water—hot water.

No attempt was made to close the artificial opening. No pain ensued, though there was considerable febrile reaction the first twenty-four hours. The bladder was washed out with hot boric acid water once daily for one week. The greater portion of the urine has drained per vaginam. Micturition per urethram was performed at irregular intervals, and was free from pain. The urine gradually showed less and less of the ropy characteristics, the mucous appearance and the admixture of blood almost disappeared. The patient got up and was about the house, being, but for the dribbling of the urine, perfectly comfortable. The fistula was purposely left open, and the bladder thus allowed to drain away its urine and be at perfect rest. This condition remained, the urine passing largely per vaginam, till two years after the second operation, when the patient died from an extension of the disease involving the entire bladder.

The second patient is a young colored domestic, æt. 17, who came into the hospital about four weeks ago complaining of frequent micturition, incontinence of urine, amenorrhoea and some elevation of the temperature and increased frequency of the pulse. The urethra and bladder were very sensitive, and great pain was complained of on the introduction of the catheter. The urine contained mucus contaminated with blood. The patient was placed on the following treatment: Acidi boracici, dr. i.; tinct. hyoscyami, oz. ss.; infs. buchu, oz. iss. M. S. One teaspoonful every three hours. A few days later conjoined manipulation discovered a tumor about the size of an almond situated on the posterior vesical wall. The bladder was ordered washed out every other day with a hot saturated solution of boric acid, and the above prescription was continued. The patient improved rapidly, and was able to leave the hospital about a month after her admission, the tumor having entirely disappeared. Had the tumor not yielded so promptly to treatment I would have scraped it out.

I had two other cases of this trouble under my care; one a lady from Kentucky, the other from this city. Both were placed on the same treatment given the second case here related, and both made a good recovery.

We have three important points brought out by these cases: the diagnosis of papillomatous growths of the bladder, early in their history; the frequency and pathological significance of papilloma of the bladder; the best method of dealing with them. In diagnosing this trouble, we should remember that papilloma of the bladder always causes, even in its very early development, some hemorrhage. The differential diagnosis between renal and vesical hemorrhage is not always easy. When the growth or growths are too small to be made out by physical examination, the evidence must rest upon the seat of localization of the pain, etc., the pressure of vesical tenesmus, much mucus in the urine, the bright red color of the urine, and the comparative small amount of urine and the absence of tube-casts. The microscope will aid when the villous formations break down.

Papillomas of the bladder are, without doubt, more frequent than is generally supposed. In the region of the bladder these tumors take on a soft variety which, if situated externally and at some accessible point, possess but little pathological significance. They do not have a tendency to glandular affection or dissemination, and the infiltration of the adjacent mucous membrane and sub-mucous tissue is probably inflammatory. On the part of the bladder, hemorrhage is the first, the chief, and the most constant symptom and result. It is the hemorrhage which, in the interior parts, gives to these growths their most important pathological significance. Complete removal is not always easy, and if not done recurrence is sure. The possibility of an epithelioma developing from a papilloma must not be forgotten. Reliable authorities say there is no relief for this affection; that it will destroy life in two years. It is doubtful whether this view is strictly correct. In the majority of cases it is unquestionably true, but some, there is reason to believe, are curable. These growths are, then, in their anatomical and microscopical features, innocent, but on account of their location, their inaccessibility, the serious and persistent hemorrhages which they create, their strong tendency to return through imperfect or incomplete removal, and finally, a possibility of a degeneration into epithelioma, they are in a general sense malignant.

If dealt with surgically, shall they be attacked through the urethra, or shall vaginal cystotomy be performed? The necessary amount of dilatation of the urethra is easily accomplished, but is apt to unduly stretch the urethral and cystic muscular fibres, and to lacerate some of their tissues, or the surrounding parts. The urethra and bladder have been torn from their surrounding attachments by forcible dilatation. Not only temporary but permanent incontinence of urine has followed these manipulations—results which time, medicine and surgery have failed to remedy. The alleged advantages of this method of operation for the removal of morbid growths and foreign bodies of any considerable size from the bladder, do not compensate for the disadvantages. The operation of vaginal cystotomy is easy of execution, and if properly done, a safe procedure. It admits of a thorough exploration of the bladder and its consequent contents, and affords ample opportunity for surgical procedures. As such, it should largely supersede exploration and manipulative procedures through the urethra. In cystotomy we should be careful not to cut too low down into the neck of the blader, or too high up around the cervix uteri, or too far on either side, wounding the ureters. The endoscope is a failure as a means of diagnosis, though theoretically it would seem of value. The finger is too short for use through the urethra. One-half of it is consumed by this organ, and the other half could reach only an inch or an inch and a half into the bladder. The urethra and the neck of the bladder so constrict on the finger as to quickly benumb it and curtail its power as an instrument for operation.

